# The Impact of Insulin Pump Therapy on Glycemic Control and Acute Diabetes Complications in Type 1 Diabetes: Real-World Evidence From a Single Center

**DOI:** 10.7759/cureus.93242

**Published:** 2025-09-25

**Authors:** Osama Saad BinDajam, Samia Abdallah Bokhari, Muneera Abd Ulmalik Alshareef, Turki Abdul Aziz Al-Harthi, Shadia Talat Howladar, Nouf Abdulkarim Alshehri, Shahad Jamil Ashgar, Razan Z Alnugali, Fahad Saud Albogami, Haidar Mohammed Haidar Alshamrani

**Affiliations:** 1 Diabetes and Endocrinology, King Fahad Armed Forces Hospital, Jeddah, SAU; 2 Adult Diabetes and Endocrinology, King Fahad Armed Forces Hospital, Jeddah, SAU; 3 Pediatric Endocrinology, King Fahad Armed Forces Hospital, Jeddah, SAU; 4 Adult Diabetes and Endocrinology, King Abdullah Medical Complex, Jeddah, SAU; 5 Family Medicine and Diabetes Management, King Fahad Armed Forces Hospital, Jeddah, SAU

**Keywords:** abbott freestyle libre 2 continuous glucose monitoring, advanced hybrid closed-loop (ahcl) algorithm, diabetes complications, glycemic control, impact, insulin pump therapy, minimed™ 780g system, type 1 diabetes

## Abstract

Background and objectives

The adoption of insulin pumps in diabetes management has seen a significant rise globally, offering promising advancements in glycemic control. Despite this progress, notable disparities remain in the utilization of this technology across different populations. We hypothesized that insulin pump therapy improves glycemic control and reduces acute complications compared to multiple daily injection (MDI) therapy. This study aimed to assess the impact of insulin pump therapy on glycemic control and acute diabetes complications among patients with type 1 diabetes mellitus at King Fahad Armed Forces Hospital (KFAFH), Jeddah. We sought to compare patient outcomes before initiating insulin pump therapy (while on MDI insulin therapy), using Abbott FreeStyle Libre 2 continuous glucose monitoring (CGM), to their results after transitioning to insulin pump therapy for six months. The findings will contribute to understanding the effectiveness of this technology in a clinical setting and addressing gaps in its adoption.

Methods

A cross-sectional study was conducted among patients with type 1 diabetes mellitus aged seven years and older who attended the Diabetes and Endocrine Department at KFAFH, Jeddah. Data were collected using a structured survey.

Results

The study included 24 patients with type 1 diabetes mellitus, with a mean age of 22.79 ± 8.86 years and a mean BMI of 24.35 ± 4.96. The mean age at diagnosis was 12.25 ± 5.29 years. Most patients (n = 22, 91.7%) used the MiniMed™️ 780G system, which employs an advanced hybrid closed-loop (AHCL) algorithm. All participants underwent carbohydrate-counting sessions prior to the initiation of insulin pump therapy. After six months of insulin pump therapy, significant improvements (p<0.05) were observed in hemoglobin A1C (HbA1c) or glucose management indicator (GMI) levels, Percentage of time spent in target blood glucose range (TIR) per day, Percentage of time below target glucose range (TBR) per day, percentage of time very low below target glucose range per day, Frequency of hypoglycemia per week, diabetic ketoacidosis (DKA) admissions, and meal time flexibility. The findings will contribute to understanding the effectiveness of this technology in a clinical setting and addressing gaps in its adoption

Conclusions

Insulin pump therapy is associated with improved and sustained glycemic control in patients with type 1 diabetes mellitus. Achieving optimal blood glucose levels is critical for reducing the risk of diabetes-related complications and lowering mortality rates. These improvements significantly enhance the quality of life and reduce the economic burden associated with diabetes management.

## Introduction

Diabetes mellitus is widely recognized as a significant and growing public health concern globally, impacting both emerging and industrialized nations and carrying substantial economic and social implications, posing significant health challenges for affected individuals [1]. According to the World Health Organization (WHO), approximately 422 million people globally have diabetes mellitus, with the majority residing in low- and middle-income countries. Diabetes mellitus accounts for approximately 1.5 million fatalities annually. Its incidence and prevalence have been progressively rising over recent decades [2] and are projected to rank as the sixth leading cause of death by 2030 [3].

Maintaining optimal glycemic control is crucial in managing individuals with type 1 diabetes mellitus, as it significantly reduces the risk of severe long-term complications [4]. A 1% reduction in hemoglobin A1C (HbA1c) levels correlates with a 21% decrease in diabetes-related mortality, a 37% reduction in microvascular complications, and a 14% reduction in myocardial infarction [5]. Hypoglycemia presents a major challenge in the effective management of type 1 diabetes mellitus. Its incidence has risen over the past decade due to the increased use of insulin therapy [6]. Severe hypoglycemia can result in critical complications, including stroke, acute heart failure, heart attack, and arrhythmias [7]. These complications highlight the importance of addressing quality-of-life (QOL) concerns as they profoundly influence patients' perceptions of their condition and overall well-being.

Despite numerous advancements, achieving optimal glycemic control remains a significant challenge for many individuals with type 1 diabetes mellitus [8]. Poorly managed diabetes often results in reduced quality of life, emotional distress, and chronic microvascular and macrovascular complications [9]. Insulin pump therapy offers a more physiological insulin delivery method compared to multiple daily injections (MDI). It often improves glycemic control without increasing hypoglycemia risk and provides greater flexibility for individualized diabetes management [9].

The effectiveness of diabetes treatment is increasingly linked to improved metabolic outcomes, patient satisfaction, and QOL [9]. Key components of QOL for individuals with type 1 diabetes mellitus include interpersonal relationships, social performance, and emotional well-being. Adherence to strict guidelines - such as insulin administration, dietary restrictions, physical activity, and regular blood glucose monitoring - can lead to significant psychological strain. Patients frequently experience feelings of guilt over inadequate glycemic control, fear of weight gain, and persistent worry about hypoglycemia. Over time, these factors can result in emotional exhaustion, reduced motivation, and a lack of adherence to treatment regimens. Diabetes distress, characterized by the psychological strain and anxiety associated with managing this complex condition, is a significant aspect of the diabetes experience and should be distinguished from depression [10].

## Materials and methods

**Figure 1:**
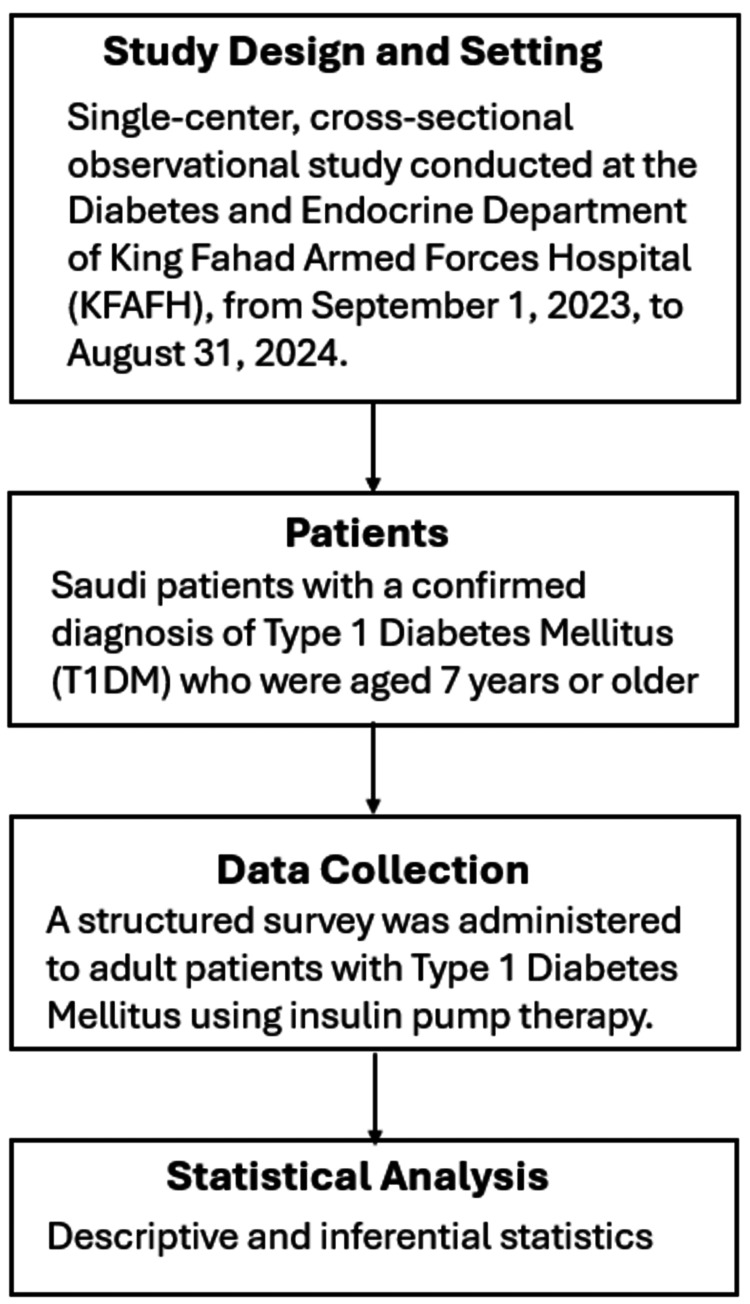
Flowchart illustrating the study design, participant selection, data collection process, and statistical analysis

The overall study process is summarized in Figure 1.

Study design and setting

This was a single-center, cross‑sectional observational study conducted at the Diabetes and Endocrine Department of King Fahad Armed Forces Hospital (KFAFH), Jeddah, Saudi Arabia, over 12 months from September 1, 2023, to August 31, 2024. KFAFH serves a diverse population of military personnel and their dependents, providing specialized diabetes care, including advanced insulin pump therapy. The study enrolled Saudi patients with a confirmed diagnosis of type 1 diabetes mellitus who were aged seven years or older. Diagnosis of type 1 diabetes mellitus was established based on clinical presentation, requirement for continuous insulin therapy since diagnosis, and laboratory evidence. Patients with clinical or biochemical features suggestive of type 2 diabetes mellitus or latent autoimmune diabetes in adults (LADA) were excluded. Patients were also excluded if they were non-Saudi nationals, younger than seven years, pregnant during the study period, or had discontinued or experienced insulin pump device failure before enrollment. A total of 24 patients fulfilled the inclusion criteria and were enrolled in the study.

Data collection and study variables

A structured survey was administered to adult patients with type 1 diabetes mellitus using insulin pump therapy. Data were collected at the Diabetes and Endocrine Department of KFAFH, Jeddah, Saudi Arabia. Eligible patients were identified from the hospital diabetes registry and were approached during routine outpatient clinic visits. All data were collected in a standardized manner, through face-to-face interviews, and supplemented from patients' electronic medical records, by trained clinical staff to ensure accuracy, reproducibility, and consistency.

The survey was designed to capture a comprehensive set of variables spanning demographic, anthropometric, clinical, insulin pump-related, and lifestyle data. Demographic and anthropometric data included age, gender, educational attainment, and current BMI based on WHO criteria [11]. Clinical variables encompassed age at diagnosis of type 1 diabetes mellitus and total duration of the disease. Insulin pump-related data included the type and model of the device, the date of initiation, the name of the institution where pump therapy was initiated, and whether the patient had received structured carbohydrate counting education before pump initiation. The structured carbohydrate counting education is delivered by certified diabetes educators and dietitians. It consists of individualized sessions covering identification of carbohydrate-containing foods, estimation of carbohydrate portions, adjustment of insulin-to-carbohydrate ratios, and use of correction factors. This education is typically provided before initiating insulin pump therapy and reinforced during subsequent follow-up visits.

Clinical outcome measures were obtained from patients’ medical records and included the most recent HbA1c value and/or corresponding Glucose Management Indicator (GMI), as well as continuous glucose monitoring (CGM) metrics such as the latest values of time in range (TIR; 70-180 mg/dL), time below range (TBR; <70 mg/dL), and time very low below range (<54mg/dL). The burden of hypoglycemia was assessed via patient self-report and classified by both frequency (none, one to two times per month, one to two times per week, three to four times per week, five to six times per week, or daily) and severity. Hypoglycemia can be classified into three levels: level 1 (mild), defined as blood glucose <70 mg/dL but ≥54 mg/dL; level 2 (moderate), defined as blood glucose <54 mg/dL; and level 3 (severe), characterized by severe cognitive or physical impairment requiring external assistance [12,13]. Earlier classifications have also considered blood glucose <40 mg/dL as a definition of severe hypoglycemia [14]. Frequency of hypoglycemia unawareness was also self-reported using the same frequency categories.

Acute diabetes-related complications were assessed by recording the number of diabetic ketoacidosis (DKA) admissions during the preceding six months. Lifestyle-related variables included patient-reported changes in mealtime flexibility following insulin pump initiation (categorized as improved vs. not improved), as well as changes in total daily insulin dose before and six months after initiation of insulin pump therapy.

Ethical considerations

Ethical approval for this study was obtained from the Hospital Research Ethical Committee at King Fahad Armed Forces Hospital, Jeddah, Saudi Arabia. Written informed consent was obtained from all participants or their legal guardians before their inclusion in the study.

Statistical analysis

Descriptive Statistics

Descriptive statistics were used to summarize the participants’ demographic and clinical characteristics. Categorical variables are presented as frequencies and percentages (n, %). Continuous variables are reported as means and standard deviations (mean ± SD) for normally distributed data, and as medians with interquartile ranges (median [IQR]) for skewed or non-normally distributed data, as appropriate.

Inferential Statistics

The normality of continuous data was assessed using the Kolmogorov-Smirnov test, which confirmed that the data were normally distributed. Consequently, parametric tests were applied to continuous variables. Specifically, paired samples t-tests were used to compare glycemic control parameters before and after six months of insulin pump therapy. These included comparisons of the total daily insulin dose (TDD), last HbA1c or GMI, and CGM metrics such as TIR, TBR, 54-69 mg/dL, and time very low below range (<54 mg/dL).

For categorical outcomes, appropriate non-parametric tests were applied based on the nature of the variables. The Wilcoxon signed-rank test was used for comparing ordinal variables with multiple categories, including the frequency of mild hypoglycemia (54-69 mg/dL), moderate to severe hypoglycemia (<54 mg/dL) episodes, and DKA admissions (past six months). In contrast, McNemar’s test was applied to dichotomous outcomes such as mealtime flexibility (poor vs. good) and hypoglycemia unawareness (present vs. absent). However, McNemar’s test could not be calculated for hypoglycemia unawareness due to zero counts in one category post-intervention. A two-tailed p-value of less than 0.05 was considered statistically significant. All statistical analyses were performed using IBM SPSS Statistics, version 27.0.1 (IBM Corp., Armonk, NY).

## Results

Baseline demographic and clinical characteristics of the study participants

The study included 24 participants, with a slight predominance of females (54.2%) over males (45.8%). The mean age of the participants was 22.79 years (SD = 8.86), indicating a relatively young cohort. Regarding age distribution, 29.2% of participants were under 18 years, 37.5% were between 18 and 25 years, 25.0% were aged 26-35 years, and 8.3% were >35 years. The average BMI was 24.35 kg/m² (SD = 4.96), placing the group within the normal to slightly overweight range. Based on BMI classification, 12.5% of participants were underweight (BMI <18.5 kg/m²), 33.3% had a normal BMI (18.5-24.9 kg/m²), 41.7% were overweight (BMI 25-29.9 kg/m²), and 12.5% were obese (BMI ≥30 kg/m²). In terms of educational background, a majority of participants (66.7%) had attained a university-level education, while smaller proportions had completed secondary (16.7%), intermediate (8.3%), or primary school education (8.3%). These demographic and anthropometric characteristics suggest that the sample was relatively well-educated, young, and mostly within a healthy or slightly elevated BMI range, which may positively influence their capacity for self-management and responsiveness to advanced diabetes technologies such as insulin pump therapy (Table 1).

**Table 1:** Demographic and anthropometric characteristics of the study participants (N = 24) SD: standard deviation; IQR: interquartile range; BMI: body mass index

Variables		N	%	Mean	SD	Median	IQR
Gender	Female	13	54.2%				
	Male	11	45.8%				
Age, years				22.79	8.86	21.00	16.50-27.50
Age groups	<18	7	29.2%				
	18-25	9	37.5%				
	26-35	6	25.0%				
	>35	2	8.3%				
BMI, kg/m^2^				24.35	4.96	25.45	19.75-28.72
BMI category	Underweight (<18.5 kg/m²)	3	12.5%				
	Normal (18.5-24.9 kg/m²)	8	33.3%				
	Overweight (25-29.9 kg/m²)	10	41.7%				
	Obese (≥30.0 kg/m²)	3	12.5%				
Patient education level	Intermediate school	2	8.3%				
	Primary school	2	8.3%				
	Secondary school	4	16.7%				
	University	16	66.7%				

Clinical characteristics and insulin pump-related information among the study participants

**Figure 2:**
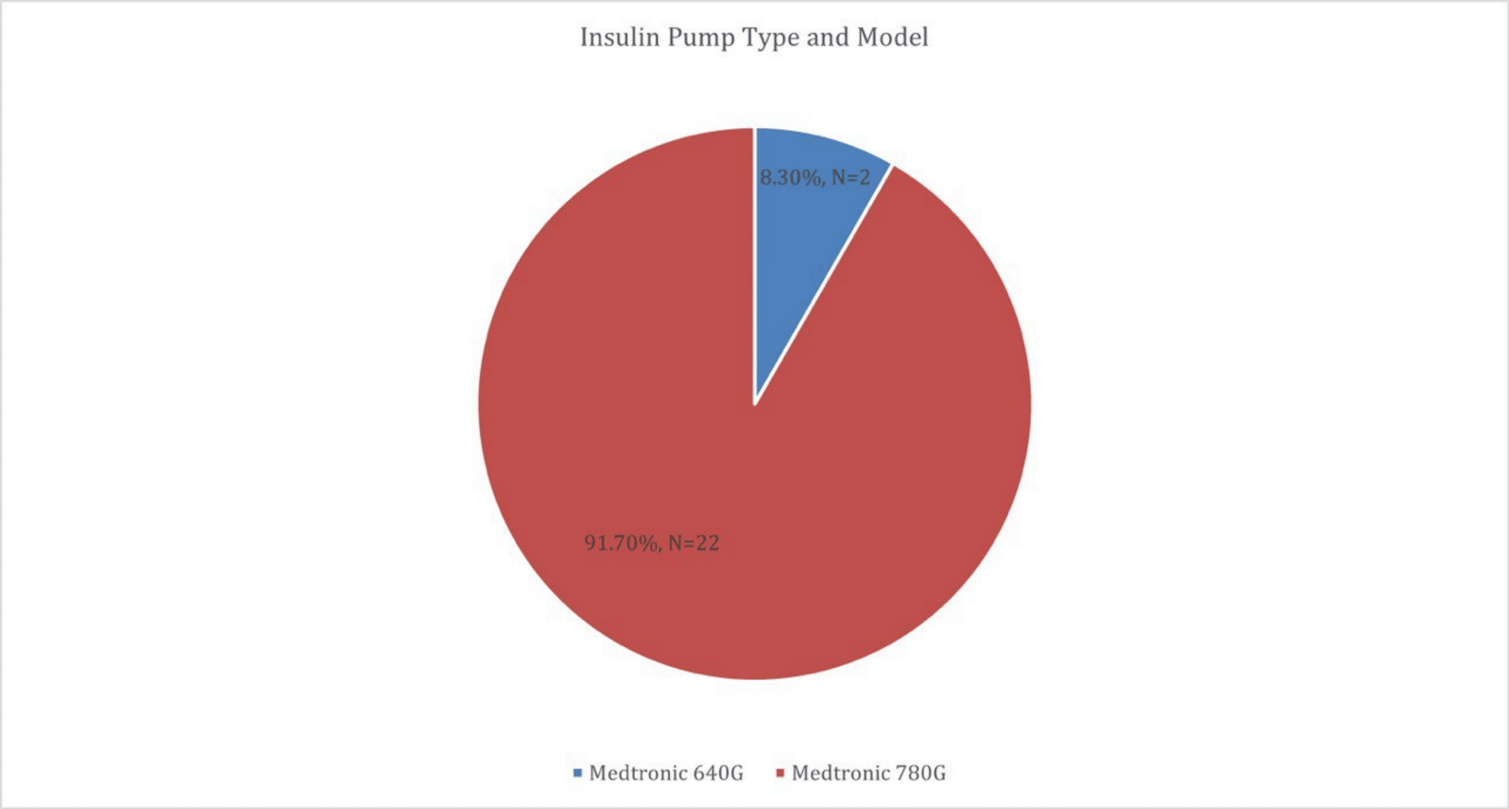
Distribution of insulin pump types and models among study participants

The clinical profile of the 24 participants revealed a mean age at diagnosis of type 1 diabetes of 12.25 years (SD = 5.29), with 29.2% diagnosed before the age of 10, 54.2% between ages 10 and 18, and 16.7% after 18. The average duration of diabetes was 10.54 years (SD = 8.38), with an even split: 50.0% had been living with diabetes for less than 10 years, while the other 50.0% had a disease duration of 10 years or more, reflecting a cohort with a broad range of diabetes experience. In terms of insulin pump usage, the vast majority of participants (91.7%) were using the MiniMed™ 780G system, while 8.3% were using the MiniMed™ 640G sensor augmented pump system. All pumps were installed at KFAFH in Jeddah, ensuring a standardized clinical setting for device initiation. Notably, all participants (100.0%) had received prior counseling in carbohydrate counting, indicating a uniformly high level of exposure to structured diabetes self-management tools before transitioning to insulin pump therapy. These findings highlight a cohort with substantial clinical experience, diverse disease duration, and strong foundational knowledge in diabetes care, which likely supports successful adaptation to advanced diabetes technologies (Table 2, Figure 2).

**Table 2:** Clinical characteristics and insulin pump-related information among the study participants SD: standard deviation; IQR: interquartile range

Variables		N	%	Mean	SD	Median	IQR
Age at diagnosis, years				12.25	5.29	12.00	8.00-16.50
Age	<10	7	29.2%				
	10-18	13	54.2%				
	>18	4	16.7%				
Diabetes duration, years				10.54	8.38	9.00	4.00-15.00
Duration of diabetes	<10	12	50.0%				
	≥10	12	50.0%				
Insulin pump device used	MiniMed™ 640G system	2	8.3%				
	MiniMed™ 780G system	22	91.7%				
Name of the institution where pump therapy was initiated	KFAFH-Jeddah	24	100.0%				
Has the patient had carb-counting sessions before installing the insulin pump?	Yes	24	100.0%				

Comparison of glycemic control parameters before and after insulin pump therapy

The glycemic control parameters of the 24 participants were comprehensively evaluated while on MDI insulin therapy using the Abbott FreeStyle Libre 2 CGM system and after six months of insulin pump therapy. The mean TDD of insulin decreased from 53.00 units (SD = 30.73) to 47.50 units (SD = 27.00), reflecting a downward trend in insulin requirements, although this reduction was not statistically significant (p = 0.173). A marked and statistically significant improvement was observed in glycemic markers, with the mean HbA1c or GMI level dropping from 8.52% (SD = 2.68) to 6.78% (SD = 0.73) (p = 0.002), indicating enhanced long-term glucose control and reduced risk of diabetes-related complications.

**Figure 3:**
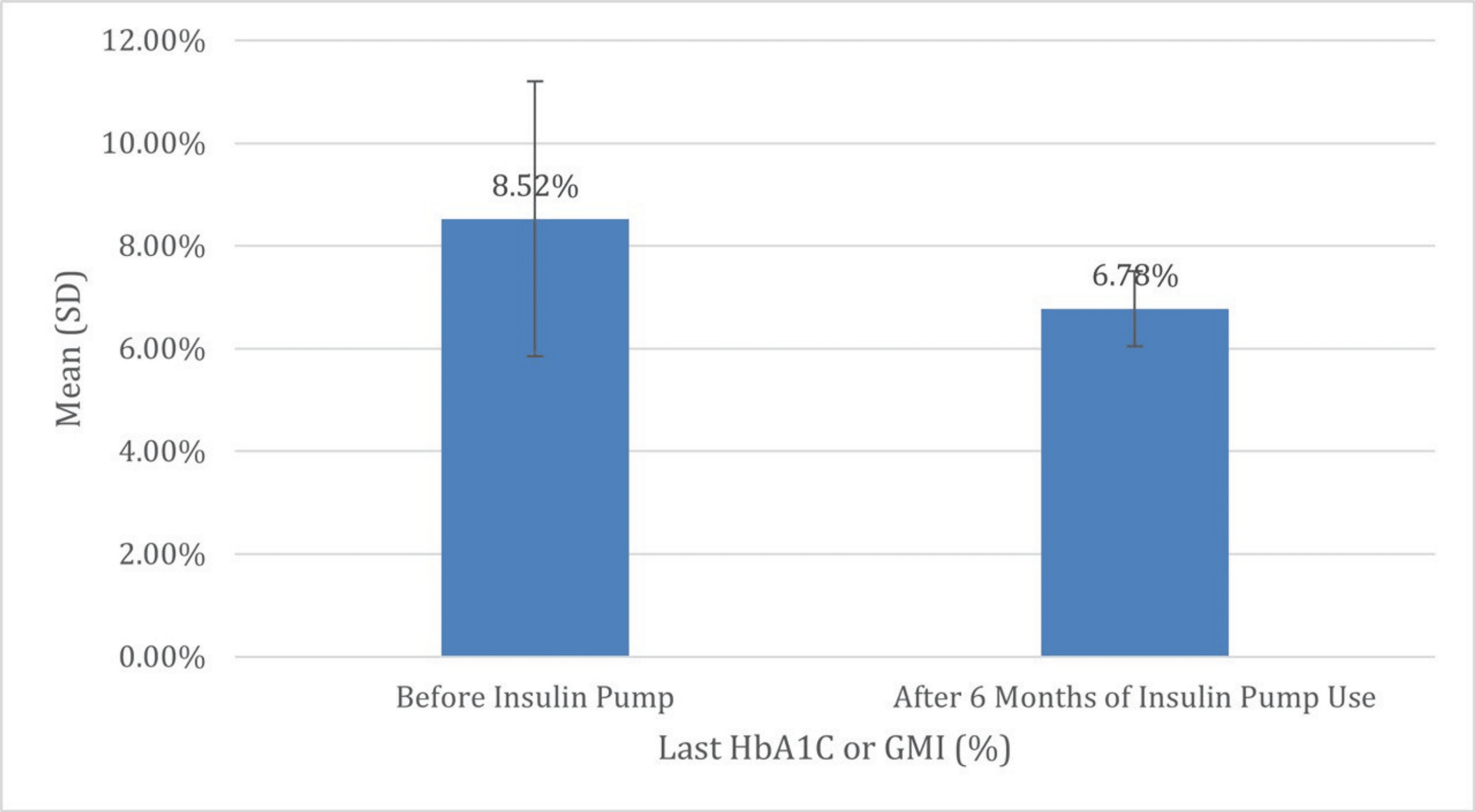
Comparison of HbA1c and/or GMI before and after six months of insulin pump therapy Figure 2 demonstrates a statistically significant reduction in HbA1c and/or GMI values after six months of insulin pump therapy, with mean levels decreasing from 8.52% to 6.78% (p = 0.002). This notable decline suggests improved long-term glycemic control and reflects enhanced treatment adherence and efficacy associated with pump use compared to MDI HbA1c: hemoglobin A1c; GMI: glucose management indicator; SD: standard deviation; MDI: multiple daily injections

**Figure 4:**
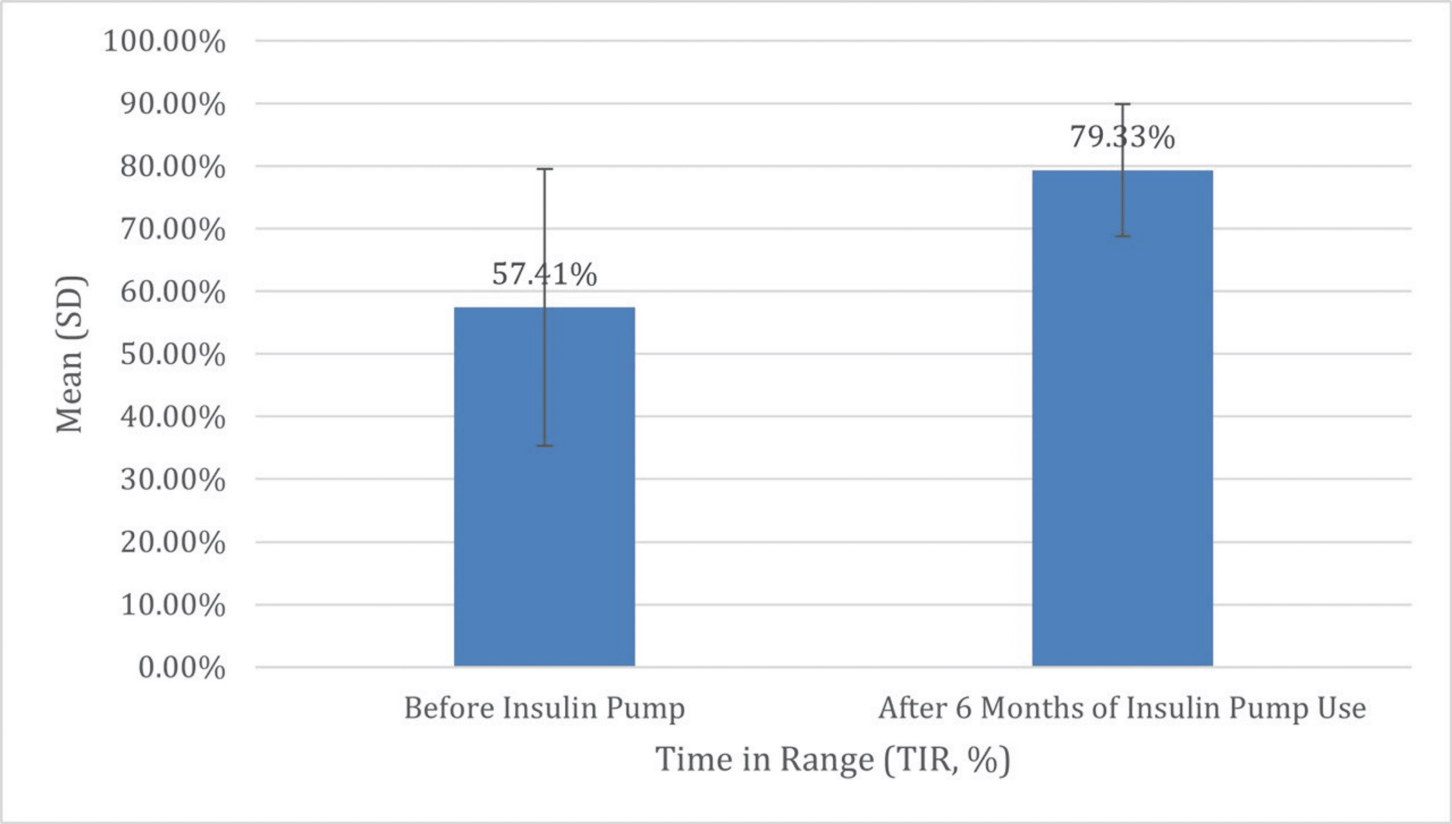
Comparison of the last TIR before and after six months of insulin pump therapy Figure 3 highlights a substantial increase in the mean of the last TIR, improving from 57.41% (SD = 22.10) before insulin pump initiation to 79.33% (SD = 10.57) after six months of therapy (p<0.001), indicating greater overall glucose stability TIR: time in range; SD: standard deviation

**Figure 5:**
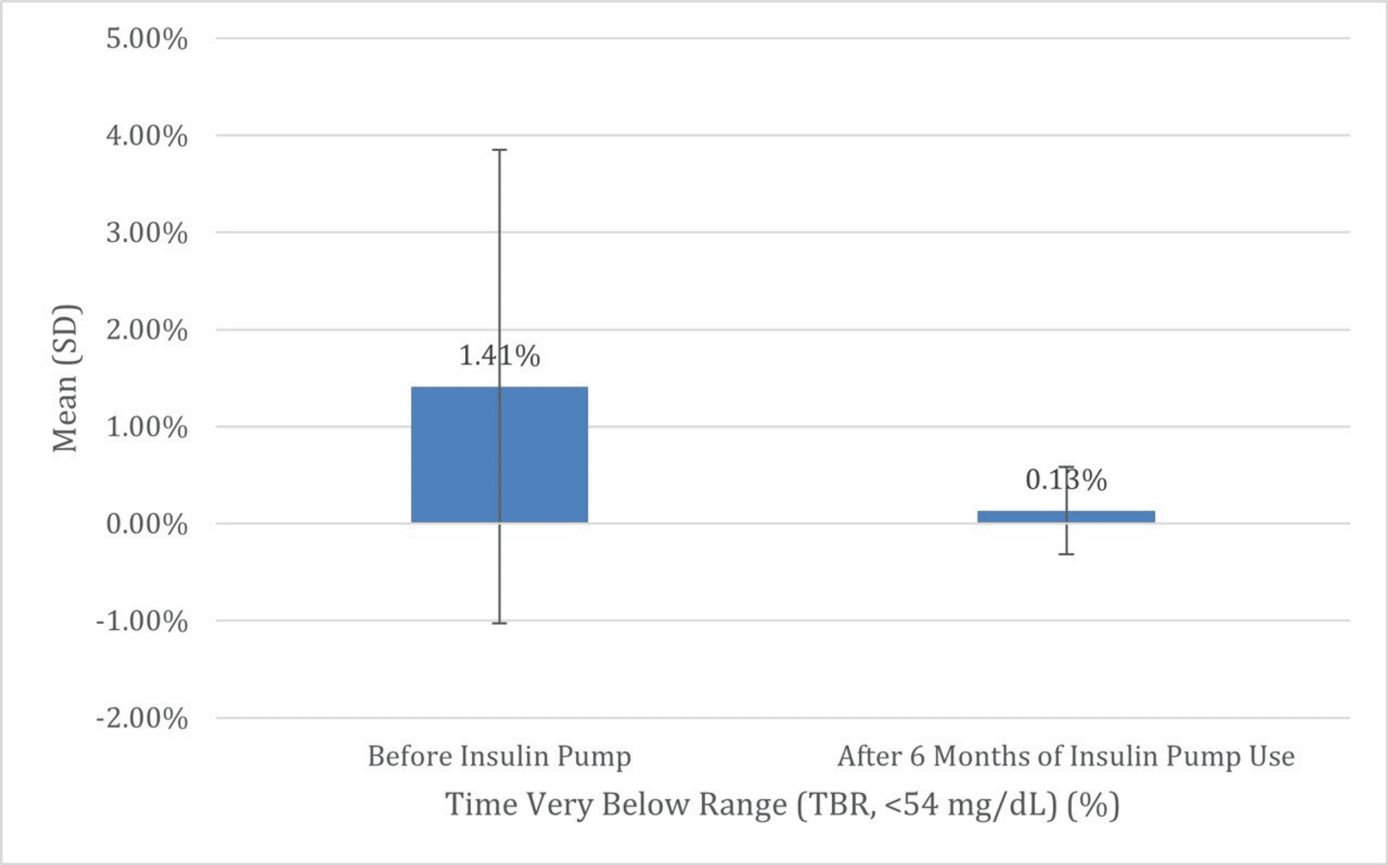
Comparison of the mean of the last percentage of the very low TBR before and after six months of insulin pump therapy Figure 4 shows a significant drop in the mean of the last percentage of time participants experienced the very low TBR (glucose <54 mg/dL), from 1.41% to 0.13% (p = 0.027). This finding confirms the safety advantage of insulin pump therapy by reducing the risk of potentially dangerous hypoglycemic events TBR: time below range; SD: standard deviation

**Figure 6:**
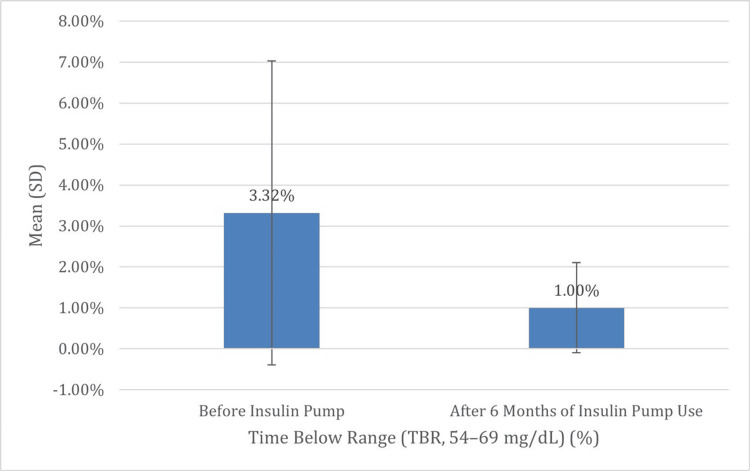
Comparison of the mean of the last percentage of TBR before and after six months of insulin pump therapy Figure 5 illustrates a marked reduction in mild hypoglycemic episodes, with the mean of the last percentage of time spent in the low TBR (54–69 mg/dL) decreased from 3.32% (SD = 3.71) to 1.00% (SD = 1.10) (p = 0.004). This reduction underscores the benefit of advanced insulin delivery systems in minimizing the frequency of hypoglycemia, which is a major barrier to optimal glycemic control TBR: time below range; SD: standard deviation

**Figure 7:**
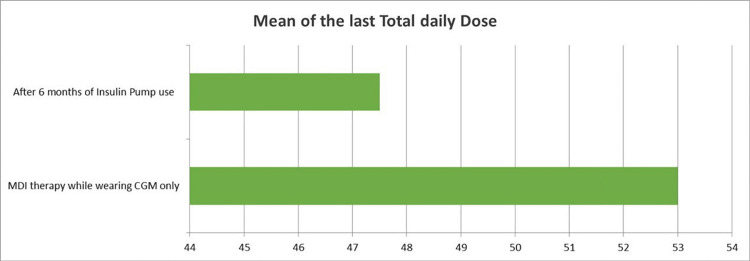
The effect of using insulin pump therapy on the mean of the last TDD of insulin Figure 6 illustrates that the mean TDD of insulin before initiating insulin pump therapy was 53 IU, whereas after six months of insulin pump therapy, it decreased to 47.5 IU. This reduction was not statistically significant (p = 0.173). TDD: total daily dose; MDI: multiple daily injections; CGM: continuous glucose monitoring

Moreover, there was a significant increase in TIR, with the mean percentage rising from 57.41% (SD = 22.10) to 79.33% (SD = 10.57) (p < 0.001), highlighting improved overall glucose stability. Hypoglycemia also significantly improved following pump initiation: The mean percentage of time spent in the very low TBR (below 54 mg/dL) was reduced from a mean of 1.41% (SD = 2.44) to 0.13% (SD = 0.45) (p = 0.027), and the mean percentage of time spent in the low TBR (54-69 mg/dL) decreased from 3.32% (SD = 3.71) to 1.00% (SD = 1.10) (p = 0.004). These changes collectively indicate that insulin pump therapy led to meaningful improvements in glycemic outcomes by reducing HbA1c, increasing time in range, and minimizing both the frequency and severity of hypoglycemia, all of which contribute to safer and more effective diabetes management (Table 3, Figures 3-7).

**Table 3:** Comparison of glycemic control parameters before and after insulin pump therapy for six months ^t^Paired Sample t-test. ^*^Significant at p<0.05

Variable		Before insulin pump				After 6 months of insulin pump use				T	df	95% CI		P-value^t^
		Mean	SD	Median	IQR	Mean	SD	Median	IQR			Lower	Upper	
Glycemic control parameters	The last total daily dose (TDD) of insulin (units)	53	30.73	46	31.00-60.00	47.5	27	37	27.40-65.90	1.407	22	-2.268	11.851	0.173
	Last HbA1C and/or GMI (%)	8.52%	2.68%	7.30%	6.60%-10.90%	6.78%	0.73%	6.80%	6.40%-7.00%	3.498	21	0.70%	2.77%	0.002^*^
	Last time in range (TIR, %)	57.41%	22.10%	60.50%	52.00%-71.00%	79.33%	10.57%	79.00%	75.00%-87.50%	-4.999	21	30.701%	-12.662%	<0.001^*^
	Last percentage of time below target glucose range (TBR, 54–69 mg/dL) (%)	3.32%	3.71%	1.00%	0.00%-6.00%	1.00%	1.10%	1.00%	0.00%-2.00%	3.203	21	0.85%	3.97%	0.004^*^
	Last percentage of time very low below target glucose range (TBR, <54 mg/dL) (%)	1.41%	2.44%	0.50%	0.00%-1.00%	0.13%	0.45%	0.00%	0.00%-0.00%	2.378	21	0.16%	2.39%	0.027^*^

Comparison of hypoglycemia frequency, DKA admissions, and mealtime flexibility before and after insulin pump therapy

This analysis offers a comprehensive evaluation of clinical and lifestyle outcomes before and after six months of insulin pump therapy among 24 participants. A significant reduction in the frequency of mild hypoglycemic episodes (blood glucose 54-69 mg/dL) was observed post-therapy (p<0.001), with the percentage of individuals reporting no such episodes increasing from 13.0% to 50.0%. Notably, high-frequency occurrences - such as daily and five to six times per week - were entirely eliminated following pump initiation. Similarly, the incidence of moderate to severe hypoglycemia (<54 mg/dL) declined significantly (p = 0.007), with 75.0% of participants reporting no episodes, up from 56.5% before pump use. Importantly, no participant experienced moderate or severe hypoglycemia on a daily or near-daily basis after starting pump therapy.

Although not statistically analyzed due to its rarity, hypoglycemia unawareness showed complete resolution, with all participants (100.0%) reporting full awareness after pump therapy, compared to one individual affected prior. Additionally, the rate of DKA admissions in the past six months was significantly reduced (p = 0.025); the proportion of participants without any DKA episodes rose from 79.2% to 95.8%. Most strikingly, mealtime flexibility underwent a dramatic transformation (p < 0.001), with the percentage of participants reporting improved mealtime flexibility increasing from 4.2% before pump use to 95.8% afterward.

**Figure 8:**
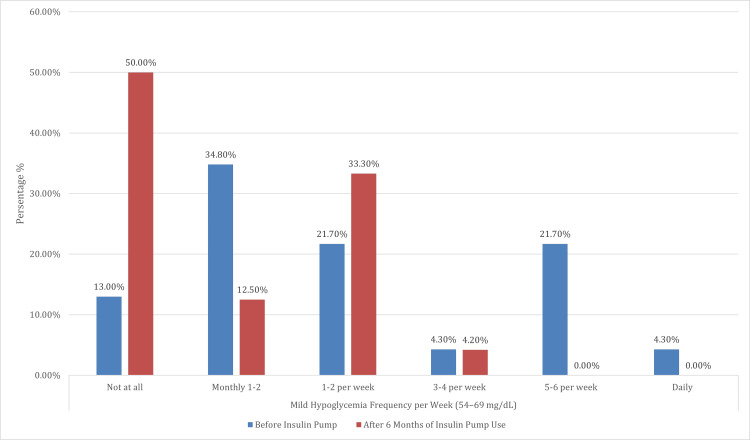
Comparison of mild hypoglycemia episodes (54–69 mg/dL) before and after six months of insulin pump therapy This figure illustrates the distribution of mild hypoglycemia episodes (blood glucose 54–69 mg/dL) among participants before and after six months of insulin pump use. A significant improvement was observed (p<0.001), with the proportion of participants reporting no mild hypoglycemic episodes increasing markedly from 13.0% to 50.0%. Conversely, higher frequency episodes substantially declined: daily and 5–6 times per week events, which were reported by 4.3% and 21.7% of participants, respectively, at baseline, were completely eliminated after insulin pump initiation. These findings suggest that insulin pump therapy effectively reduced the burden and frequency of mild hypoglycemia in this cohort

**Figure 9:**
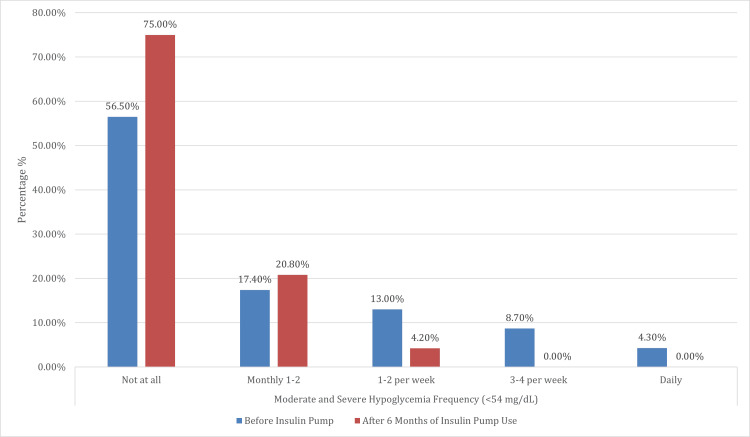
Comparison of moderate and severe hypoglycemia frequency (<54 mg/dL) before and after six months of insulin pump therapy This figure illustrates the distribution of moderate and severe hypoglycemia episodes (moderate hypoglycemia when blood glucose is <54 mg/dL, while severe hypoglycemia is <40 mg/dL) among participants before and after six months of insulin pump use. A significant improvement was observed (p = 0.007), with the proportion of participants reporting no such episodes increasing from 56.5% to 75.0%. In contrast, the frequency of higher-risk hypoglycemia events declined markedly. Daily episodes, as well as those occurring 3–4 times per week, were entirely eliminated post-insulin pump therapy. The proportion of participants experiencing episodes 1–2 times per week also decreased from 13.0% to 4.2%. These results indicate that insulin pump therapy significantly reduced the occurrence of moderate to severe hypoglycemia, contributing to enhanced patient safety and glycemic stability

**Figure 10:**
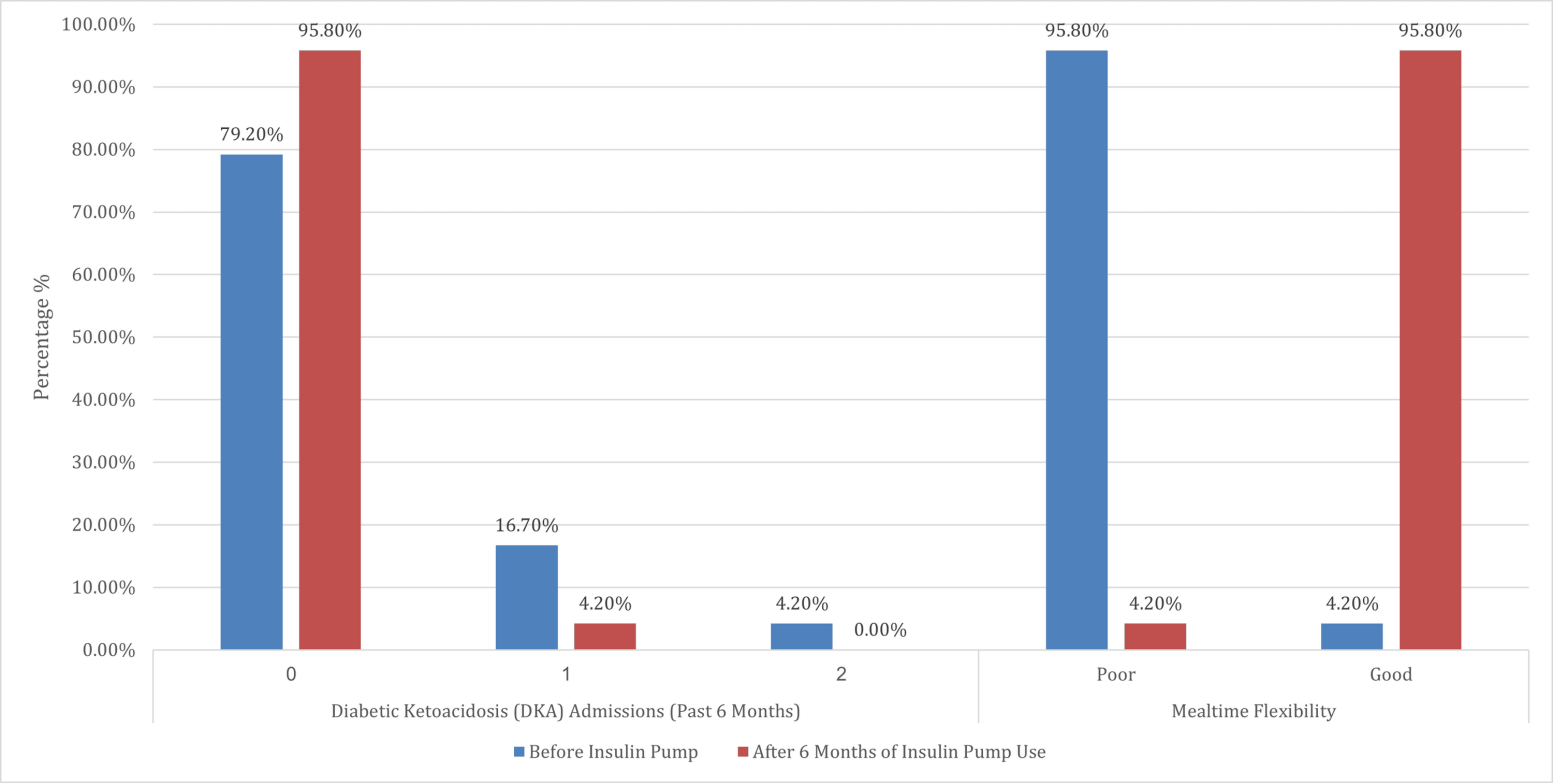
Comparison of DKA admissions and mealtime flexibility before and after six months of insulin pump therapy This figure illustrates the changes in DKA admissions over the past 6 months and perceived mealtime flexibility before and after six months of insulin pump therapy. The proportion of participants with no DKA episodes increased significantly from 79.2% to 95.8%, while those reporting one or more admissions declined correspondingly. Notably, no participants reported more than one DKA episode after pump initiation. This reduction (p = 0.025) reflects the improved metabolic stability and reduced risk of acute complications associated with insulin pump use Regarding mealtime flexibility, a dramatic improvement was observed following pump therapy. Before its use, 95.8% of participants reported poor flexibility in meal timing, whereas after six months, the same percentage (95.8%) reported good flexibility (p<0.001). This shift underscores the significant lifestyle benefit conferred by insulin pump therapy, allowing for greater freedom in daily routines and meal planning without compromising glycemic control DKA: diabetic ketoacidosis

Overall, these findings highlight the considerable clinical and lifestyle advantages of insulin pump therapy, including a reduction in the frequency and severity of hypoglycemia, decreased DKA incidence, elimination of hypoglycemia unawareness, and substantially enhanced mealtime flexibility, reflecting both improved metabolic control and quality of life (Table 4) (Figures 8-10).

**Table 4:** Comparison of hypoglycemia frequency, DKA admissions, and mealtime flexibility before and after insulin pump therapy ^W^Wilcoxon signed-rank test. ^N^McNemar’s test. ^*^Significant at p<0.05 DKA: diabetic ketoacidosis; CGM: continuous glucose monitoring

Variables		For 6 months before insulin pump therapy, while on MDI therapy and wearing a CGM only		After 6 months of insulin pump therapy		Test statistics	Standard error	P-value^W/N^
		N	%	N	%			
Mild hypoglycemia frequency per week (54–69 mg/dL)	Not at all	3	13.0%	12	50.0%	11.000	26.113	<0.001^*^
	Monthly 1-2	8	34.8%	3	12.5%			
	1-2 per week	5	21.7%	8	33.3%			
	3-4 per week	1	4.3%	1	4.2%			
	5-6 per week	5	21.7%	0	0.0%			
	Daily	1	4.3%	0	0.0%			
Moderate and severe hypoglycemia frequency (moderate hypoglycemia is <54 mg/dL, while severe hypoglycemia is <40 mg/dL)	Not at all	13	56.5%	18	75.0%	<0.001	8.292	0.007^*^
	Monthly 1-2	4	17.4%	5	20.8%			
	1-2 per week	3	13.0%	1	4.2%			
	3-4 per week	2	8.7%	0	0.0%			
	Daily	1	4.3%	0	0.0%			
Hypoglycemia unawareness	Not at all	23	95.8%	24	100.0%			-
	Monthly 1-2	1	4.2%	0	0.0%			
DKA admissions (past 6 months)	0	19	79.2%	23	95.8%	<0.001	3.354	0.025^*^
	1	4	16.7%	1	4.2%			
	2	1	4.2%	0	0.0%			
Mealtime flexibility	Not Improved	23	95.8%	1	4.2%			<0.001^*^
	Improved	1	4.2%	23	95.8%			

## Discussion

The current investigation included a cross-sectional study of 24 individuals diagnosed with type 1 diabetes mellitus. 50% of the patients had been diagnosed with diabetes for 10 or more years, while the remaining 50% had been diagnosed for less than 10 years. Most patients (91.7%) utilized the MiniMed™ 780G system, whereas a minority (8.3%) employed the MiniMed™ 640G sensor-augmented pump system. Before insulin pump therapy, all patients underwent carb-counting sessions.

The use of insulin pump therapy for six months significantly improved glycemic control. The mean HbA1c (and/or GMI) decreased from 8.52 ± 2.68 to 6.78 ± 0.73 (p = 0.002), while the mean TIR increased from 57.41 ± 22.10% to 79.33 ± 10.57 (p<0.001). These findings align with those of the American Diabetes Association (ADA) (2023), which demonstrated improved glycemic outcomes and reduced severe hypoglycemia rates with insulin pump therapy compared to MDI. Consistent with our findings, ADA (2023) reported improved glycemic outcomes in individuals on insulin pump therapy [15]. While hypoglycemia was a major adverse effect of intensified insulin therapy in the Diabetes Control and Complications Trial (DCCT) [4], data suggest that insulin pumps may reduce the rates of severe hypoglycemia compared with MDI [15].

Type 1 diabetes mellitus and its complications impose substantial financial burdens. The annual cost for individuals with type 1 diabetes mellitus ranges from $9,000 without complications to $32,000 with severe complications [16,17]. However, the mean TDD of insulin before initiating insulin pump therapy was 53 IU, while the mean TDD after six months of insulin pump therapy was 47.5 IU. This difference was not statistically significant (p = 0.173). According to the Saudi FDA price list, one international unit of Insulin Glargine 300 IU/mL costs 0.158 Saudi Riyal (SAR) per unit ($0.042 per IU), and Insulin Aspart, typically used for the insulin pump reservoir or pre-meal bolus, costs 0.126 SAR per unit ($0.034 per IU). Our study found that the mean daily insulin dose was lower on insulin pump therapy (47.5 IU per day vs. 53 IU per day). If all patients were on the basal-bolus regimen before using the insulin pump (50% basal/50% bolus), the mean daily basal insulin dose was 26.5 IU, and the mean daily bolus insulin dose was 26.5 IU. Consequently, the daily cost of insulin per patient reduced from 7.526 SAR ($2.00) to 5.985 SAR ($1.59), and the annual cost per patient decreased from 2,746.99 SAR ($731.81) to 2,184.53 SAR ($581.97), resulting in a direct annual savings of 562.46 SAR ($149.84) per patient, or approximately 13,499.04 SAR ($3,596.22) for all patients in the study [18].

The frequency of mild hypoglycemia episodes (54-69 mg/dL) significantly decreased after six months of pump therapy (p<0.001). Moderate or severe hypoglycemia episodes (<54 mg/dL and <40 mg/dL, respectively) also decreased significantly (p = 0.007). While DKA incidence significantly declined in our study (from five to one admissions; p = 0.025), this finding contrasts with some prior studies, such as that by Alshami et al. (2021), and may reflect recent advances in pump technology and patient education [19]. A notable 95.8% of patients reported improved mealtime flexibility with insulin pump therapy. This is consistent with studies, such as those by Almogbel et al. (2020), which demonstrated improved metabolic control and reduced HbA1c levels with pump therapy [20]. Insulin pumps’ ability to deliver precise basal and bolus doses enhances metabolic stability and patient satisfaction.

While the present study found complete resolution of hypoglycemia unawareness and a significant reduction in DKA admissions (p = 0.025), the latter is consistent with findings by Karges et al., which reported reduced DKA incidence with pump therapy [21]. Conversely, a meta-analysis by Misso et al. observed no reduction in non-severe hypoglycemia with pump use, highlighting variability in outcomes depending on patient populations and study designs [22]. Emerging technologies, such as fully closed-loop systems, have the potential to further reduce glycemic variability, hypoglycemia, and patient burden. Improvements in CGMs and insulin delivery algorithms could yield substantial economic and health benefits [23].

Insulin pump therapy significantly improves glycemic control, reduces hypoglycemia frequency, and enhances the quality of life for individuals with type 1 diabetes mellitus [24]. Although it markedly impacts DKA admissions, its cost-effectiveness and potential to improve long-term outcomes make it a valuable tool in diabetes management. Continued advancements in technology and patient education will likely enhance these benefits further. Before 1993, most studies suggested an increased risk of DKA with insulin pump use [25]. However, due to advances in pump technology and structured patient education, DKA has become a less prevalent complication with insulin pump therapy. Recently, large population-based observational studies in Europe on patients with type 1 diabetes mellitus showed a decreased risk of DKA with insulin pump use [21,22].

The local cost of one-day admission for one patient in the medical ward under the Department of Diabetes and Endocrinology in King Fahad Armed Forces Hospital, Jeddah, Saudi Arabia, is 2,290 SAR per day ($610.67 per day). DKA admission typically takes an average of three days to resolve, depending on multiple factors. According to our study, the total number of DKA admissions in the past six months while on insulin pump therapy, compared to the six months before using insulin pump therapy, showed a decrease in admissions (1 vs. 5) (p = 0.025). Thus, reducing the number of DKA admissions from five to one resulted in a total reduction of hospital days from 15 to three days. Therefore, calculating the cost reduction for 12 days of admission would yield total savings of 27,480 Saudi Riyals ($7,328).

Emerging treatment options and technologies have the potential to reduce the cost of type 1 diabetes mellitus. Access to continuous glucose monitors and insulin pump technologies can minimize the risk of complications and emergencies, thereby reducing average lifetime costs by 14% and yielding a benefit-cost ratio of 1.5. Additionally, drug therapies and screening can help identify individuals who have not yet been clinically diagnosed, providing treatments that delay the onset of complications and prevent emergencies at diagnosis. A reduction of HbA1c by 1.0%-1.5% and an improvement in TIR to 65% or more - without significant safety risks - could result in an annual economic impact of $5-$10B in the US, depending on the level of efficacy achieved. Fully closed-loop pumps, which can achieve TIRs of 95% and minimize user burden, could yield an annual economic impact of $18B in the US [23,26].

In our study, most patients (n = 23, 95.8%) showed a significant improvement (p<0.001) in their ability to adjust mealtime flexibility following the implementation of insulin pump therapy for six months. A study conducted in Saudi Arabia by Almogbel et al. (2020) indicated that pump therapy was linked to reduced HbA1c levels, suggesting enhanced metabolic control compared to individuals using MDI [20]. This corroborates the findings of a prior study [27]. Similarly, a separate trial demonstrated that insulin pump therapy effectively and safely improved diabetes management, reducing insulin requirements while sustaining improvements in lipid profile and blood pressure among individuals with type 2 diabetes [28].

Another study verified that insulin pump therapy offers potential advantages for diabetes management [29]. In a cluster randomized trial examining the efficacy of insulin pumps versus MDI for adults with type 1 diabetes, both groups underwent comparable training in flexible insulin treatment. The study revealed that both the insulin pump and MDI groups experienced significant and enduring reductions in HbA1c levels and rates of severe hypoglycemia, clinically significant outcomes. Additionally, the study documented that the inclusion of pump therapy did not significantly improve educational outcomes in terms of glycemic control or hypoglycemia prevention [30]. There is evidence indicating that a controlled carbohydrate diet can have beneficial effects on metabolic regulation and can help reduce HbA1c levels. Furthermore, a controlled carbohydrate diet may decrease the occurrence of hypoglycemia [31]. All participants in the present study had received structured carbohydrate-counting education before pump initiation.

While not statistically analyzed due to the rarity of baseline hypoglycemia unawareness, all patients reported full awareness post-pump therapy, suggesting a possible benefit. Additionally, there was a significant difference (p = 0.025) in DKA admissions for the six-month periods before and after using insulin pump therapy. The findings presented here are consistent with the conclusions published by Karges et al., who found that pump therapy was linked to a reduced occurrence of DKA and severe ketoacidosis compared to injectable therapy [21]. Conversely, a meta-analysis conducted by Misso et al. examined 23 studies involving 976 participants with Type 1 diabetes who were randomly assigned to either insulin pump therapy or multiple insulin injections.

The analysis revealed that there were no apparent disparities between the two interventions in terms of non-severe hypoglycemia [22]. However, individuals using insulin pumps experienced a reduction in severe hypoglycemia. The findings of this investigation were consistent with those of the current study. Furthermore, the REPOSE trial, which observed adults with type 1 diabetes, discovered that the utilization of insulin pump therapy was linked to a higher occurrence of hypoglycemia episodes compared to MDI throughout a two-year follow-up period [30]. Although insulin pump therapy can more accurately imitate the natural insulin release in the body, it can also provide more effective insulin delivery to tissues and reduce the likelihood of hypoglycemia episodes [32,33].

Strengths and limitations

The main strength of the present study lies in its assessment of the impact of insulin pump therapy on blood glucose control and acute diabetes complications. This was achieved through the analysis and six-month follow-up of patients with type 1 diabetes mellitus who used insulin pump therapy, comparing their results with those from the same patients during a period when they were on MDI insulin therapy. CGM while on MDI insulin therapy was performed using the Abbott FreeStyle Libre 2 system. Although the reduction in insulin usage was not statistically significant, it translated to modest annual cost savings, further supported by the substantial savings from avoided DKA admissions. However, there are several limitations to this study. The design was cross-sectional, which may introduce bias. Additionally, the small sample size, lack of a controlled group, and short follow-up (six months) are significant limitations. The study also did not address factors such as diabetes education, patient motivation, and family support, which are crucial in influencing the risk of hypoglycemia and ketoacidosis [34,35].

## Conclusions

The current study demonstrates that insulin pump therapy is associated with improved and sustainable glycemic control across all age groups with type 1 diabetes. Achieving effective glycemic control is critical to preventing both macrovascular and microvascular complications, as well as reducing mortality rates. Beyond clinical outcomes, these improvements also enhance quality of life and reduce the economic burden of the disease. Importantly, the findings highlight the need for further research to evaluate healthcare services and to identify factors that influence diabetes control. Such evidence can inform policies and practices aimed at optimizing diabetes management and improving patient outcomes. However, given the relatively small sample size, these conclusions should be interpreted with caution when generalizing to broader populations.
